# Does Variation among Provincial Drug Formulary Antimicrobial Listings in Canada Influence Prescribing Rates?

**DOI:** 10.1371/journal.pone.0107515

**Published:** 2014-09-09

**Authors:** Shiona K. Glass-Kaastra, Rita Finley, Jim Hutchinson, David M. Patrick, Karl Weiss, John Conly

**Affiliations:** 1 Centre for Food-borne, Environmental and Zoonotic Infectious Diseases, Public Health Agency of Canada, Guelph, Ontario, Canada; 2 Division of Medical Microbiology, Island Medical Program, University of British Columbia, Victoria, British Columbia, Canada; 3 British Columbia Centre for Disease Control, Vancouver, British Columbia, Canada and School of Population and Public Health, University of British Columbia, Vancouver, British Columbia, Canada; 4 Department of Infectious Diseases and Microbiology, Hôpital Maisonneuve-Rosemont, University of Montreal, Montreal, Québec, Canada; 5 Department of Medicine, University of Calgary, Calgary, Alberta, Canada; Department of Microbiology, Immunology and Infectious Diseases, University of Calgary, Calgary, Alberta, Canada; Department of Pathology and Laboratory Medicine, University of Calgary, Calgary, Alberta, Canada, and Synder Institute for Chronic Diseases, University of Calgary, Calgary, Alberta, Canada; Royal College of Surgeons, Ireland

## Abstract

**Background:**

The financial accessibility of antimicrobial drugs to the outpatient community in Canada is governed at the provincial level through formularies. Each province may choose to list particular drugs or impose restriction criteria on products in order to guide prescribing and/or curtail costs. Although changes to formularies have been shown to change patterns in the use of individual products and alter costs, no comparison has been made among the provincial antimicrobial formularies with regards to flexibility/stringency, or an assessment of how these formularies impact overall antimicrobial use in the provinces.

**Objectives:**

To summarize provincial antimicrobial formularies and assess whether their relative flexibility/stringency had a statistical impact upon provincial prescription volume during a one year period.

**Methods:**

Provincial drug plan formularies were accessed and summarized for all prescribed antimicrobials in Canada during 2010. The number of general and restricted benefits for each plan was compiled by antimicrobial classification. Population-adjusted prescription rates for all individual antimicrobials and by antimicrobial class were obtained from the Canadian Integrated Program for Antimicrobial Resistance Surveillance. Correlations between the number of general benefits, restricted benefits, and total benefits with the prescription rate in the provinces were assessed by Spearman rank correlation coefficients.

**Results:**

Formularies varied considerably among the Canadian provinces. Quebec had the most flexible formulary, offering the greatest number of general benefits and fewest restrictions. In contrast, Saskatchewan's formulary displayed the lowest number of general benefits and most restrictions. Correlation analyses detected a single significant result; macrolide prescription rates decreased as the number of general macrolide benefits increased. All other rates of provincial antimicrobial prescribing and measures of flexibility/stringency revealed no significant correlations.

**Conclusions:**

Although antimicrobial formulary listings are used to guide prescribing rates within a province, our analysis of one year's data of the impact of the antimicrobial formulary structure did not correlate with antimicrobial prescribing rates, and other factors are likely to be at play.

## Introduction

Although physician visits and hospital services in Canada are covered by universal insurance programs administered by the provinces, prescription drug benefits are not covered by a single source [1]. From the perspective of outpatient drug prescriptions, there are 10 separate systems in Canada; one for each province [1]. Each province reviews the recommendations for approval by the Common Drug Review (CDR), which is the national body tasked with evaluating the clinical evidence for new drug products and new applications of current products, and the cost-effectiveness of the new product/new applications [2]. The provinces then make their own decisions to permit or deny reimbursement for the product through their respective formularies. Furthermore, provinces have the option to list products as either general benefits (available to all plan members by prescription) or as a restricted benefit (requiring additional information and/or paperwork before prescriptions may be reimbursed). As each province is governed independently in this respect, formularies may be quite different and may have differing time intervals from CDR approval to formulary decision. These differences are expected to affect patterns of drug use among the provinces.

A method that has been employed to direct prescribing and potentially contain costs is to list products as restricted benefits, rather than offering all drugs as general benefits. Restrictions may include 1) requiring additional clinical details confirming a diagnosis, or 2) requiring the physician to write a code on the prescription itself that is submitted electronically by the pharmacist filling the prescription; without this code, the prescription will not be reimbursed. Although termed differently among the provinces (e.g. limited use drugs in Ontario, exceptional medications in Québec) [3], [4], each of these programs increases the burden of work placed upon the prescriber in order for the patient to access the product and receive financial reimbursement. Knowledge of these criteria is required by the prescriber, since pharmacists do not have the jurisdiction to interchange antimicrobials (although they may interchange generic and brand name products of the same drug formulation). As such, if the restricted use criteria are not fulfilled, patients are either 1) required to pay for their antimicrobial out-of-pocket, 2) return to the prescribers for a new prescription, or 3) leave the prescription unfilled. These requirements are therefore expected to reduce the use of the restricted drugs, particularly for non-approved indications [5]. As such, provinces with a high number of restricted benefits may be expected to have a lower prescribing rate than provinces with more general benefits.

Due to the nature of antimicrobial use as an immediate and short-term treatment, it has been suggested that restrictions do not lead to reduced overall antimicrobial use, but rather lead to an increase in the use of an alternate general benefit antimicrobial, a “squeezing of the balloon” phenomenon [6]. This may not be a negative consequence, as it may be possible to employ restrictions as a guide for prescribing antimicrobials that are less likely to select for resistant isolates. Therefore, if employed in this manner, such restrictions may be seen as beneficial to the medical community as a tool for antimicrobial stewardship. However, the authors recognize that formularies are currently prepared on the basis of providing the least expensive product, which may not be the most prudent choice for antimicrobial stewardship. Therefore, in order to make this change, the financial impact of treating infections caused by antimicrobial resistant pathogens must be taken in to account (e.g., increased duration and burden of illness).

Although complete formulary comparisons have been made on the basis of the percentage of all available drugs covered [7], to the best of our knowledge, an analysis of the reimbursement status for antimicrobial drugs across the Canadian provinces has not been published. Another provincial factor expected to influence antimicrobial prescribing differences is new product review times. The time frame between a positive CDR review and the addition of a new product to a formulary may vary among the provinces. It has been suggested that this review process is comparatively expedient in Québec [8], however, no data exists which compares these wait times.

In order to have a composite picture of antimicrobial use in Canadian provinces, it was considered essential to understand the availability of these drugs as a potential driving factor for their use. Consequently, we sought to conduct a study with the following objectives:1) to summarize the benefit status of individual antimicrobial drugs among the provinces, 2) to describe requirements for reimbursement of restricted products in each province, 3) to summarize the time required for provincial review of drug products following the CDR approval, and 4) to assess the significance of the number of general benefit and restricted benefit antimicrobials in each province with the total and antimicrobial-class specific rates of provincial prescribing.

## Methods

Provincial formularies were accessed online through the health ministries in each province, and the most recent version was obtained [9]–[18]. Each formulary was searched for every antimicrobial approved in 2010 as described in Finley et al., 2013 [19], and recorded as a general benefit, a restricted benefit, or not a benefit for each province. An assumption was made that an antimicrobial was not a benefit in a given province if the product could not be found anywhere within the respective formulary. Antimicrobials were grouped into six classes (cephalosporins, macrolides, penicillins, quinolones, sulfonamides and trimethoprim derivatives, tetracyclines, others and miscellaneous antimicrobials), as defined by the World Health Organization (WHO) Anatomical Therapeutic Classification (ATC) system [20]. Reimbursement criteria for restricted benefits and the time lapse from CDR approval to listing on the provincial formularies were also obtained online from the respective health ministries [3], [4], [21]–[27].

The total antimicrobial prescriptions per 1000 individual-days in 2010 (PrIDs) in each province, as well as the PrIDs per antimicrobial class for the same year were acquired from the Canadian Integrated Program for Antimicrobial Resistance Surveillance (CIPARS) program. CIPARS tracks temporal and regional trends in antimicrobial use and antimicrobial resistance in selected species of enteric bacteria obtained from both food production and from human clinical laboratory sources [8]. CIPARS monitors oral antimicrobial use as dispensed by outpatient pharmacies throughout Canada using the IMS Health Canada dataset. Therefore, the prescription rate outcome assessed here describes all of the prescription antimicrobials obtained by patients in Canada (it does not capture the antimicrobials that are prescribed but not dispensed).

In order to assess the impact of formulary flexibility upon provincial antimicrobial prescribing, Spearman rank correlation coefficients were calculated between the number of general benefits on provincial formularies and their respective PrIDs, by antimicrobial class. Furthermore, Spearman rank correlation coefficients were calculated between the number of restricted benefits on provincial formularies and their respective PrIDs, by antimicrobial class, as a measure of the impact of formulary stringency. Finally, the overall number of general benefits (across all classes) and the overall number of restricted benefits (across all classes) were assessed for their impact upon provincial prescribing rates, again using Spearman rank correlation coefficients. All results were assessed with a p-value of 0.05. All calculations and analyses were performed using Stata/MP 12.1 (StataCorp, College Station, TX, USA).

## Results

The provincial formularies were summarized for all 40 antimicrobial drugs dispensed in Canada in 2010 by their availability as a general benefit, a restricted benefit, or not a benefit (Table 1). Six antimicrobials were not listed as benefits in any province (chloramphenicol, gatifloxacin, gemifloxacin, penicillin G, sulfadiazine, and tobramycin) (Table 1). Additionally, pivampicillin was only listed in a single province (Nova Scotia) (Table 1). The median number of total benefits (general and restricted) available was 29 (range: 26–33), while the number of general benefits was 23 (14–31), and the number of restricted benefits was 6 (1–15). The province with the strictest formulary was Saskatchewan, with 14 general benefits and 15 restricted benefits. In stark contrast, Québec had the largest number of general benefits (31) with just a single restricted product (Table 1).

**Table 1:** Provincial formulary listing status in 2011 for all antimicrobial drugs prescribed in Canada (2010).

Class^1^	Drug	AB	BC	MB	NB	NF	NS	ON	PE	QC	SK
CEF	Cefaclor		*	RE	*	*		*		*	
	Cefadroxil	RE	*	*	*		*	*		*	
	Cefixime	*	*	RE	*		*	*	*	*	RE
	Cefprozil	*		RE		RE	*	*	RE	*	RE
	Cefuroxime axetil	* 2	*	RE	*	*	*	*	RE	*	RE
	Cephalexin	*	*	*	*	*	*	*	*	*	*
MAC	Azithromycin	*	*	RE	*	*	RE	*	RE	*	RE
	Clarithromycin	*	*	RE	*	*	RE	*	RE	*	RE
	Clindamycin	*	*	*	*	*	*	*	*	*	*
	Erythromycin	*	*	*	*	*	*	*	*	*	*
	Spiramycin		*	*	*		*			*	
	Telithromycin	RE					*			*	RE
OTH	Chloramphenicol										
	Erythromycin and sulfisoxazole		*	*	*					*	
	Fosfomycin										RE
	Fusidic acid					*	*				
	Linezolid	RE	RE		RE	RE	RE	*	RE	RE	RE
	Methenamine		*		*		*				*
	Metronidazole	*	*	*	*	*	*	*	*	*	*
	Nitrofurantoin	*	*	*	*	*	*	*	*	*	*
	Tobramycin			*							
	Vancomycin	RE	RE	*	*		RE		RE	*	RE
PEN	Amoxicillin	*	*	*	*	*	*	*	*	*	*
	Amoxicillin/clavulanic acid	*	*	RE	*	*	*	*	*	*	RE
	Ampicillin	RE	*		*	*	*	*	*	*	*
	Cloxacillin	*	*	*	*	*	*	*	*	*	*
	Penicillin G										
	Penicillin V	*	*	*	*	*	*	*	*	*	*
	Pivampicillin						*				
QUI	Ciprofloxacin	RE	*	RE	RE	*	RE	RE	RE	*	RE
	Levofloxacin	RE		RE	RE	RE	RE	RE	RE	*	RE
	Moxifloxacin	RE	*	RE	*		RE	RE	RE	*	RE
	Ofloxacin	RE	*	RE		*	RE	RE	RE	*	
	Norfloxacin	*	*	RE	*	*	RE	*	RE	*	RE
SUL	Sulfadiazine										
	Sulfamethoxazole/trimethoprim	*	*	*	*	*	*	*	*	*	*
	Trimethoprim	*	*	*	*	*	*	*	*	*	*
TET	Doxycycline	*	*	*	*	*	*		*	*	*
	Minocycline	*	*	RE	*	*	*		RE	*	RE
	Tetracycline	*	*	*	*	*	*	*	*	*	*
TOTAL number of:	General benefit antimicrobials	20	28	15	27	23	15	24	23	31	14
	Restricted benefit antimicrobials	9	2	11	3	3	12	9	3	1	15
	Antimicrobials not covered	11	10	14	10	14	13	7	14	8	11
Total antimicrobial prescriptions filled per 1000 individuals (PrIDs)		1.91	1.72	1.96	1.94	2.97	2.04	1.93	2.05	1.54	2.31

1 CEF  =  cephalosporins, MAC  =  macrolides, OTH  =  others and miscellaneous antimicrobials, PEN  =  penicillins, QUI  =  quinolones, SUL  =  sulfonamides and trimethoprim derivatives, TET  =  tetracyclines.

AB  =  Alberta, BC  =  British Columbia, MB  =  Manitoba, NB  =  New Brunswick, NF  =  Newfoundland, PE  =  Prince Edward Island, ON  =  Ontario, QC  =  Québec, SK  =  Saskatchewan.

*General benefit, RE  =  restricted benefit, blank  =  not a benefit.

2 de-listed June 30^th^ 2010.

Generally, the class most affected by requirements for reimbursement was the quinolones; only British Columbia and Québec had no restricted listings for this class. However, it should be noted that levofloxacin was not listed as a benefit for British Columbia, while it was a general benefit in Québec (Table 1). The sulfonamide and trimethoprim derivative class was not affected by restrictions; however, sulfadiazine was not a benefit in any province. The specific requirements by each province for obtaining reimbursement for a restricted product are quite similar among the provinces (Table 2). In contrast, the amount of time required for review of a drug product following CDR approval vary widely; with goal times for review ranging from 6 weeks to more than 2 years (Table 2).

**Table 2:** Summary of provincial drug plan restrictions, criteria for reimbursement, and the lag time from Common Drug Review approval to provincial decision in Canadian provinces.

Provincial drug program	Name of Restriction	Process for reimbursement^1^	Review time in months^2^
Alberta Prescription Drugs	Special authorization	Written form sent by mail or fax by the physician.	4
British Columbia PharmaCare	Special authorization	Written form sent by mail or fax by the physician. Urgent requests by phone.	Standard –9 Complex –12
Manitoba Pharmacare	Exception drug	Written form sent by mail or fax. Urgent requests by phone.	3–4 (audit review)
New Brunswick Prescription drug program	Special authorization	Written form sent by mail or fax by the physician.	5.5–11 (research review)
Nova Scotia Pharmacare	Exception drug	Linezolid – Written form sent by mail or fax by the physician. Other antimicrobials – drug and diagnosis specific ‘criteria code’ written on the prescription by the physician, input by pharmacist during online request for coverage.	1.5–4
Newfoundland Prescription Drug Program	Special authorization	Written form sent by mail or fax by the physician. Additional documentation such as confirmation of diagnosis by diagnostic testing	2.5–9
Ontario Drug Benefit	Limited Use	3 digit drug and diagnosis specific ‘reason for use’ code written on the prescription by the physician. Code is input at the pharmacy during online request for coverage.	2
Prince Edward Island Pharmacare	Exceptional Drug	Written form sent by mail or fax by the physician.	11– >24
Québec Prescription Drug Insurance	Exceptional medications	Written form sent by mail or fax by the physician.	6
Saskatchewan Drug Plan	Exception drugs	Written form sent by mail or fax by the physician. Urgent requests by phone.	Not available

1 All provinces requiring form submission requested prescriber and patient identification information, the drug requested, and a justification for the request.

2 Time to review is the goal for the province, unless otherwise reported.

Correlation analyses to assess the relationship between the number of general or restricted benefits with the total and class-specific PrIDs in 2010 revealed only a single significant result. A negative correlation (−0.64) was found between the number of general macrolide benefits and the macrolide PrIDs in 2010 (p = 0.048).

## Discussion

Provincial formularies in Canada display a wide range in coverage for antimicrobial drugs. Considerable variation exists in the number of general and restricted benefits available to beneficiaries, the number of products requiring authorization for use and the means by which authorization is acquired. Antimicrobial drugs were most strictly regulated in Saskatchewan, while the least strictly regulated in Québec. Interestingly, we found no significant correlation between overall formulary flexibility/stringency and the overall provincial antimicrobial prescribing rates., Although statistically significant differences existed among the provincial prescribing rates [28] in 2010, these rates were not affected by the structure of the respective formularies in our analysis for that year. At the antimicrobial class level, the only significant correlation found was for the macrolide class; for this group of antimicrobials, the number of general benefits was inversely correlated with the prescription rates among the provinces. Therefore, as the number of general benefit options increased for prescribers to choose from, the number of macrolide prescriptions in that province was reduced. This result contrasts with the idea that highly flexible formularies produce high prescribing rates through easy financial access to products.

The finding of no significant correlations among total antimicrobial prescribing rates and measures of flexibility/stringency may suggest that increasing restrictions do not impact the overall prescribing rates for antimicrobials in Canada. However, given the methodology employed with a limited analysis of a single data point in time (2010) for each province and the resultant limited overall power of our comparisons, we cannot preclude a relationship does not exist.

Studies analyzing administrative changes imposing specific restrictions to provincial formularies have been reported to be effective in changing the use of a particular drug within a province [5], [29]. It is possible that other factors such as greater emphasis and delivery of educational programs and greater adherence to treatment guidelines may have had an impact in specific provinces. It is also possible that in provinces with high rates of prescribing broad spectrum agents relative to narrow spectrum agents, new, or more demanding restrictions for these broad spectrum agents may reduce the use of these products, and ultimately, reduce overall prescribing.

The number of antimicrobial products that have restrictions differs among the provinces; however, the process to obtain reimbursement for these prescriptions is quite similar. With the exception of Ontario and a single product (linezolid) in Nova Scotia, the process for obtaining reimbursement for restricted products requires the prescriber to contact the insurance body with patient and prescriber identification, the product requested (strength, formulation, duration of treatment), as well as diagnostic information and a justification for use of the restricted product over another general benefit. In Ontario, and for linezolid reimbursement in Nova Scotia, the prescriber is required to write a criteria code on the prescription. These criteria codes are drug and diagnosis specific, and must be submitted online by the pharmacist at the time of prescription filling. It may be argued that the process is less strenuous for prescribers in Ontario, and further research may be required in order to determine whether the process difference between Ontario and other provinces has an impact on prescribing practices.

Timeliness is essential for formulary changes to be used as a tool to change prescribing of antimicrobials. The time requirements for provincial review of new products following CDR approval varies widely among the provinces. For example, the timeline goal for review in Ontario is 2 months, while Prince Edward Island reports that reviews may take more than 2 years. Therefore, significant changes in this review process may be required by a number of provinces in relation to antimicrobial products, if formularies are to be used for stewardship purposes. Given the wide variability in the provincial drug plan restrictions, criteria for reimbursement, and the lag time from CDR approval to provincial plan changes in Canadian provinces, our findings would suggest there is substantial room for improvement and harmonization of strategies between the provinces.

We acknowledge that our study has limitations. As mentioned, we have only conducted an analysis looking at the correlation among general or restricted benefits and the prescribing rates in a single year, which limits the generalizability of the findings. It is possible that year-to-year variations existed and that the chosen year was not representative of historical data. Unfortunately historical formulary data were difficult to obtain and we were unable to access any other years. However, there were no major policy changes during 2010. In addition, an assumption was made that the provincial drug plans adequately describe the availability of antimicrobial drugs to all individuals within the province, which may be an over-simplification of the system. Provincial plans are generally accessed by senior citizens and those on social assistance in Canada. Other individuals may be covered by third party plans offered by employers. Third party plans tend to mirror the provincial plans, such that changes to third party formularies often follow those changes made by the province. However, some third party plans may offer products over and above the provincial formularies, and therefore offer additional flexibility. Furthermore, some drugs with very specific indications may be purchased separately as part of disease control programs (e.g. benzathine penicillin G). As such, use of the provincial drug plans may underestimate access to antimicrobial drugs (particularly more expensive products) for those covered by more comprehensive third party plans. Furthermore, the proportion of individuals that are beneficiaries of the provincial plans may differ among provinces. For example, the province of Québec requires all citizens to have prescription drug coverage; if an individual is not covered by a third-party insurer, they are required to buy into the provincial plan. Therefore, a larger proportion of citizens may be covered by the provincial plan in Québec than in provinces where buy-in is optional for the uninsured.

In summary, formulary flexibility/stringency in Canadian provinces was not found to be associated with rates of antimicrobial prescribing in their respective provinces within the context of the methodology employed. However, we believe that restrictions may offer utility as a tool for antimicrobial stewardship to guide prescribing towards products that are less likely to select for resistant pathogens. It is acknowledged that the process by which antimicrobials are chosen and prescribed is complex, and further investigation is warranted, looking at other years and with additional analytic techniques particularly at the level of the prescribers. Moreover, the goal of reducing overall antimicrobial prescribing may be better approached through a multi-faceted and multi-modal program with one of the facets being the strategic use of an antimicrobial formulary process. Success in reducing overall prescribing or prescribing of targeted drugs has been acquired through multifaceted programs such as “Do Bugs Need Drugs?” in British Columbia [30], “Les Antibiotiques C'est Pas Automatique” in France [31], and the multipronged educational program and guidelines for antimicrobial use in Québec [8].
